# The GR-LEDGF/p75-HSP27 Axis Contributes to Cross-Resistance Between Enzalutamide and Docetaxel in Prostate Cancer

**DOI:** 10.3390/cells14191566

**Published:** 2025-10-09

**Authors:** Pedro T. Ochoa, Evelyn S. Sanchez-Hernandez, Alfonso M. Duran, Kai Wen Cheng, Joel Philip, Tise Suzuki, Julia J. Unternaehrer, Julie Dutil, Bhaskar Das, Rituparna Ganguly, Yasmine Baca, David de Semir, Charles Wang, Isaac Kremsky, Carlos A. Casiano

**Affiliations:** 1 Center for Health Disparities and Molecular Medicine, Department of Basic Sciences, Loma Linda University School of Medicine, Loma Linda, CA 92350, USA; pedroochoa@students.llu.edu (P.T.O.); aduran@llu.edu (A.M.D.); kaiwencheng@llu.edu (K.W.C.); joelphilipm@gmail.com (J.P.); tsuzuki@students.llu.edu (T.S.); junternaehrer@llu.edu (J.J.U.); 2 Department of Pathology and Human Anatomy, Loma Linda University School of Medicine, Loma Linda, CA 92350, USA; 3 Urology Department, Loma Linda University Medical Center, Loma Linda, CA 92354, USA; 4 University of Puerto Rico Comprehensive Cancer Center, San Juan, PR 00921, USA; jdutil@cccupr.org; 5 Department of Pharmaceutical Sciences, School of Pharmacy and Pharmaceutical Sciences, University at Buffalo, Buffalo, NY 14215, USA; bhaskard@buffalo.edu; 6 Caris Life Sciences, Phoenix, AZ 85040, USA; rganguly@carisls.com (R.G.); ybaca@carisls.com (Y.B.); ddesemir@carisls.com (D.d.S.); 7 Department of Basic Sciences, Center for Genomics, Loma Linda University School of Medicine, Loma Linda, CA 92350, USA; chwang@llu.edu; 8 Department of Basic Sciences, Division of Biomedical Engineering Sciences, Loma Linda University School of Medicine, Loma Linda, CA 92350, USA; ikremsky@llu.edu; 9 Loma Linda University Cancer Center, Loma Linda, CA 92350, USA; 10 Department of Medicine, Division of Rheumatology, Loma Linda University School of Medicine, Loma Linda, CA 92350, USA

**Keywords:** docetaxel, drug cross-resistance, enzalutamide, glucocorticoid receptor, HSP27, LEDGF/p75, prostate cancer, RNA sequencing

## Abstract

An emerging challenge in prostate cancer (PCa) treatment is the development of drug cross-resistance, wherein resistance to enzalutamide (ENZ), an androgen receptor signaling inhibitor (ARSI), also confers resistance to subsequent ARSI and docetaxel (DTX) treatments. The mechanisms underlying this drug cross-resistance remain unclear. Through RNA sequencing, we identified 93 overlapping differentially expressed genes (DEGs) in ENZ- and DTX-resistant PCa cells. Among the DEGs, *HSPB1*, which encodes heat shock protein 27 (HSP27), emerged as a key gene of interest. HSP27 is a known target of lens epithelium-derived growth factor p75 (LEDGF/p75), a transcription coactivator regulated by glucocorticoid receptor (GR). Both GR and LEDGF/p75 are overexpressed in advanced PCa and promote drug resistance. HSP27 was overexpressed in ENZ and DTX cross-resistant PCa cell lines and its expression was decreased upon GR or LEDGF/p75 silencing. ChIP sequencing confirmed GR binding at the *HSPB1* promoter. Pharmacological targeting of HSP27 in drug-resistant cells reduced proliferation, clonogenicity, and tumorsphere formation, and restored sensitivity to ENZ and DTX. Notably, high transcript expression of a GR-LEDGF/p75-HSP27 gene panel correlated with worse overall survival in PCa patients (n = 4259). These findings identified this axis as a driver of PCa drug cross-resistance and promising therapeutic target for overcoming treatment failure.

## 1. Introduction

Prostate cancer (PCa) continues to be a major cause of cancer-related deaths globally, with a 30% survival rate once patients develop metastasis [1,2,3]. Although androgen deprivation therapy (ADT) initially elicits a positive response, patients with recurrent PCa often progress to metastatic castration-resistant prostate cancer (mCRPC), an incurable form characterized by disease progression despite castrated testosterone levels [4,5]. The current standard of care for mCRPC entails a combinatorial or sequential therapy of ADT, androgen receptor signaling inhibitors (ARSI, e.g., enzalutamide, apalutamide, darolutamide, and abiraterone acetate), and taxane chemotherapy with docetaxel (DTX) or cabazitaxel (CBZ) [6]. In addition, the recent FDA-approved prostate-specific membrane antigen radioligand therapy (PSMA-RLT) for mCRPC has been considered as a game changer given its promising results in effectively targeting bone metastasis [7]. Unfortunately, PSMA-RLT remains limited in availability and is ineffective in patients with treatment-emergent neuroendocrine PCa (t-NEPC) due to downregulation of PSMA expression [8]. t-NEPC is a lethal subtype of PCa that arises in about 20–30% of patients receiving androgen-targeting therapies and exhibits resistance to both ARSIs and taxane chemotherapy through androgen-independent signaling pathways [9]. Nonetheless, none of these treatments are curative since prostate tumors can develop therapy resistance through various mechanisms [10].

A well-established mechanism of ARSI resistance in mCRPC is the reactivation of AR signaling, either through upregulation of AR itself, activation of AR co-regulators, AR mutations that modify function, or increased expression of AR splice variants such as AR-V7, which is associated with resistance to ARSIs [11,12,13,14]. On the other hand, AR blockade with ARSIs can lead to upregulation and activation of glucocorticoid receptor (GR), a member of the nuclear steroid receptor family that shares structural and functional similarities with AR [15,16,17,18,19]. For instance, prostate tumors treated with enzalutamide (ENZ) display GR overexpression and activation, promoting ARSI resistance through reactivation of a subset of AR-target genes due to AR-GR transcriptomic redundancy, thereby effectively bypassing AR blockade [15,16,17,18,19,20,21,22,23]. AR-driven adenocarcinoma may also show lineage plasticity, evolving into therapy resistant t-NEPC in subsets of patients that have received prolonged treatment with ARSI [9,24]. t-NEPC is likely driven by GR since it is promoted by ONECUT2 (OC2), a master transcription factor that upregulates GR, leading to the transcription of AR-target genes [25].

Emerging evidence supports the development of cross-resistance among distinct ARSIs and to subsequent use of DTX in mCRPC, thereby further limiting the treatment options and effective sequence [26]. While GR is well established as an antagonist of ARSI therapy, our group and others have also shown that it contributes to PCa chemoresistance through protein–protein interactions with β-catenin, and upregulation of pro-survival oncoproteins such as clusterin, lens epithelium-derived growth factor of 75 kDa (LEDGF/p75), Bcl-2 and Bcl-xL [21,27,28,29,30,31]. Moreover, as a target gene of GR [31], LEDGF/p75 functions as a transcription co-activator that interacts with several transcription factors, including GR itself [30,31]. LEDGF/p75, encoded by the *PSIP1* gene, enhances cancer cell survival under various stress conditions- such as chemotherapy, radiation, and oxidative stress-by upregulating genes involved in antioxidant defense, DNA repair, and pro-survival signaling [32,33]. It is frequently overexpressed in PCa and other malignancies and has been associated with several aggressive tumor phenotypes, including enhanced DNA repair linked to R-loop resolution, angiogenesis, proliferation, migration, clonogenicity, tumorsphere formation, and resistance to multiple chemotherapeutic agents [32,33]. Importantly, LEDGF/p75 has been shown to bind stress response elements and heat shock elements in the promoter regions of several stress survival genes, including the gene encoding heat shock protein 27 (HSP27), and to stimulate the transcriptional activation of these genes [34,35,36,37,38,39,40]. LEDGF/p75-mediated regulation of HSP27 in PCa cells has been reported by our group and others [36,41].

HSP27 is upregulated in several cancer types, including breast, prostate, ovarian cancers, and gliomas, and its elevated expression correlates with advanced disease stage and poor prognosis [42]. Mechanistically, HSP27 contributes to therapy resistance by suppressing oxidative stress responses and blocking both extrinsic and intrinsic apoptotic pathways through its interaction with either cytochrome c or the death domain-associated protein (DAXX) [43,44]. Additionally, HSP27 facilitates AR translocation from the cytoplasm to the nucleus, enhancing AR transcriptional activity [45,46,47]. The upregulation of HSP27 and other HSPs in tumor cells is considered a mechanism of cancer therapy resistance as HSPs have protein folding and chaperone functions that are critical to regulate cell proliferation, invasion, metastasis, and evasion of apoptosis [48].

GR and LEDGF/p75 are upregulated in both ENZ-resistant and DTX-resistant PCa cell lines [31] and are part of an oncogenic transcriptional network that promotes therapy resistance [30,31,32,33]. In the present study we evaluated the potential contribution of the GR-LEDGF/p75-HSP27 axis to cross-resistance between ENZ and DTX in PCa cell lines. We provide evidence that HSP27 protein expression is upregulated in both ENZ-resistant and DTX-resistant PCa cells and influenced by both GR and LEDGF/p75. In addition, targeting HSP27 with a small molecule inhibitor in combination with ENZ or DTX improves the response of drug-resistant PCa cells to these treatments. Furthermore, high tumor expression of a panel of the genes encoding GR, LEDGF/p75, and HSP27 in PCa patients correlates with unfavorable patient outcomes. Our results provide novel insights into the role of the GR-LEDGF/p75-HSP27 axis in PCa drug cross-resistance.

## 2. Materials and Methods

### 2.1. Cell Culture and Drugs

PCa cell lines PC3 (CRL-1435), DU145 (HTB-81), and LNCaP (CRL-1740) were purchased from the American Type Culture Collection (ATCC, Manassas, VA, USA) and cultured in RPMI-1640 medium (Genesee Scientific, San Diego, CA, USA, Cat# 25-506), supplemented with 10% fetal bovine serum (FBS), 1% Penicillin/Streptomycin (Corning, Glendale, AZ, USA, Cat# 30-002-CI), and Normocin 1G (Fisher Scientific, Pittsburgh, PA, USA, Cat# NC9390718). The following clinical drugs were used in our studies: docetaxel (DTX, LC Laboratories, Woburn, MA, USA, Cat# D-1000), enzalutamide (ENZ, MedChem Express, Monmouth Junction, NJ, USA, Cat# HY-70002), cabazitazel (CBZ, MedChem Express, Cat# HY-15459), and abiraterone (MedChem Express, Cat# HY-75054). DTX-resistant PC3 (PC3-DR) and DU145 (DU145-DR) cell lines were developed and characterized as previously described [30,31,32,49] and maintained in medium containing 10 nM DTX. LNCaP-ENZR, an androgen-depleted ENZ-resistant LNCaP subline, was developed in the laboratory of Dr. Carlos Diaz Osterman (University of Puerto Rico Comprehensive Cancer Center) and was kindly gifted to us. Briefly, this cell line was developed by incrementally replacing FBS with charcoal-stripped FBS (CS-FBS) in culture medium. Surviving cells resistant to androgen depletion were subsequently exposed to incremental ENZ concentrations, reaching a final concentration of 50 μM ENZ in culture. Surviving cells were maintained in medium containing 10% CS-FBS with 30 μM ENZ to avoid growth slowdown and moderate cytotoxicity caused by long-term culture in the presence of concentrations above 40 μM. Mycoplasma contamination was tested frequently in all cell lines using MycoAlert Plus assay (Lonza, Walkersville, MD, USA, Cat# LT07-218). Cell lines were authenticated by short tandem repeat profiling (Cat# ATCC-135-XV).

### 2.2. Small Interfering RNA (siRNA)-Mediated Gene Knockdown

Transient transfection of PCa cell lines was performed for 72 h using scrambled (SCR) negative control siRNA (Integrated DNA Technologies, Coralville, IA, USA, Cat# 51-01-19-09), tri-silencer siRNA targeting GR (5′-AGAAUGACCUACAUCAAAGAGCUAG, 5′-GGAUACUAUACAAG CAGAACUGAGG, and 5′-GGAGAUCAUAUAGACAA UCAAGUGC), or siRNA targeting LEDGF/p75 (5′-AGACAGCAUGAGGAAGCGAUU). siRNA transfections (25 nM or 50 nM) were performed with Interferin^®^ siRNA transfection reagent (Polyplus-Sartorius, Illkirch-Graffenstaden, France, Cat#409-01) as described previously [30,31], and protein knockdown was confirmed by immunoblotting using primary rabbit antibodies to LEDGF/p75 (Bethyl Laboratories/Fortis Life Sciences, Montgomery, TX, USA, Cat# A300-848A) and GR (Cell Signaling Technology, Danvers, MA, USA, Cat# 12041S, clone D6H2L). Glyceraldehyde-3-phosphate dehydrogenase (GAPDH, Cell Signaling Technology, Danvers, MA, USA, Cat# 2118S, clone 14C10) was used as a loading control, and primary goat HSP27 antibody (Santa Cruz Biotechnology, Dallas, TX, USA, Cat# SC-1048) was used to assess HSP27 expression. Horseradish peroxidase (HRP)-linked anti-rabbit (Cell Signaling Technology, Danvers, MA, USA, Cat# 7074S) and anti-goat (Santa Cruz Biotechnology, Dallas, TX, USA, Cat# SC-2020) secondary antibodies were used. Immunoreactive bands were detected using enhanced chemiluminescence (Fisher Scientific, Pittsburgh, PA, USA, Cat# PI34580) on autoradiography film (Midwest Scientific, Fenton, MO, USA, Cat# XC6A2). Protein bands were quantified using ImageJ software (National Institutes of Health, Bethesda, MD, USA, Fiji Version 1.44a), and relative protein expression was calculated by normalizing to GAPDH expression.

### 2.3. Cell Viability Assays

LNCaP and LNCap-ENZR cells were seeded at a density of 11,000 cells per well in triplicate wells using 96-well plates (Genesee Scientific, San Diego, CA, USA, Cat# 25-109) PC3 and PC3-DR cells were seeded at a density of 5,000 cells per well; DU145 and DU145-DR were seeded at a density of 4,000 cells per well; and 22Rv1 and 22Rv1-DR at a density of 11,000 cells per well. Cells were allowed to adhere for 24 h in their respective media in the absence of any cytotoxic drugs and subsequently treated with fresh media containing dimethylsulfoxide (DMSO, Fisher Scientific, Pittsburgh, PA, USA, Cat# PI20688) as vehicle control or with increasing concentrations of DTX (0–0.1 μM), CBZ (0–0.1 μM), ENZ (0–80 μM), or abiraterone (0–100 μM) 72 h. MTT (3-(4,5-Dimethylthiazol-2-yl)-2,5-Diphenyltetrazolium Bromide) viability assays were conducted as described previously [31]. Absorbance readings were normalized to vehicle-treated values, and the half-maximal inhibitory concentration (IC_50_) was determined in DTX-sensitive and -resistant PCa cells. To investigate the cytotoxicity of the HSP27 small molecule inhibitor J2 (MedChem Express, Monmouth Junction, NJ, USA, Cat# HY-124653), cells were treated with increasing concentrations of J2 (0–100,000 nM) for 72 h. To assess cellular sensitivity to DTX, DTX-resistant PCa cells were treated with J2 at 1 μM, 5 μM, or 10 μM in combination with increasing concentrations of DTX (0–10,000 nM). IC_50_ values were subsequently calculated to determine the degree of cellular resensitization to DTX.

### 2.4. Clonogenic Assays

PC3-DR and DU145-DR cells were seeded in 6-well plates (1000 cells/well). After 24 h, cells were exposed to either vehicle (DMSO) or J2 at concentrations of 1 μM, 5 μM, and 10 μM to assess the effects of targeting HSP27 on clonogenicity. To assess the effects of combinatory treatment with DTX and J2, cells were treated with J2 concentrations of 1 μM, 5 μM, and 10 μM in the absence or presence of DTX. Clonogenic assays were performed as described [31], with colony formation monitored for 10 days. Colony imaging was performed using a ChemiDoc™ MP Imaging System (BioRad, Hercules, CA, USA), and quantification was performed using ImageJ software with the automatic colony counting feature and identical parameters for all wells.

### 2.5. Tumorsphere Formation Assays

PCa cell-derived spheroid cultures were maintained using complete MammoCult™ medium (Stem Cell Technologies, Vancouver, BC, Canada, Cat# 05620), supplemented with hydrocortisone (0.48 μg/mL, Sigma-Aldrich, St. Louis, MO, USA, Cat# H0135), heparin (4 μg/mL, Sigma-Aldrich, St. Louis, MO, USA, Cat# H3149), and 1% penicillin/streptomycin. DU145-DR and LNCaP-ENZR cells were gently resuspended 50 times in MammoCult™ medium to ensure a single-cell suspension and seeded at 6000 cells per well in 24-well untreated plates (Genesee, Cat# 25–102) with 0.5 mL MammoCult™ medium. Tumorspheres were grown for 5 days at 37 °C/5% CO_2_ and visualized under an inverted Olympus IX70 microscope equipped with a SPOT imaging system. Quantification of total tumorsphere area, which is more indicative of proliferation capacity and response to treatment than quantification of individual spheres, was achieved with ImageJ software as described previously [28,30], using at least three independent images per individual treatment.

### 2.6. ChIP Sequencing

Publicly available ChIP datasets for *NR3C1*, the gene encoding GR, were identified using ChIP-Atlas. The search parameters used within the peak browser were the hg38 genome assembly/index, selected for *Homo sapiens*, and “ChIP: TF and others”, selected for experiment type. All cell types were considered, the peak calling threshold was set to Q < 1 × 10^−5^, and *NR3C1* was selected as the antigen. The Integrative Genomics Viewer (IGV) was used to identify peaks near the transcription start site of *HSPB1,* the gene encoding HSP27 [50]. The UCSC human genome browser (GRCh37) was also used to visualize ChIP-Atlas bigWig tracks. The GSE30623 PCa dataset was selected for further analysis based on peak, antibody, and experimental setup quality (number of replicates, treatments, and controls) [51,52]. The following PCa samples were analyzed: SRR309201 (LNCaP-1F5; GR antibody; no treatment), SRR531806 and SRR531815 (LNCaP-1F5; GR antibody; dexamethasone-treated), SRR531816 (LNCaP-1F5; GR antibody; dexamethasone- and dihydrotestosterone-treated). The GSE175482 dataset, based on ChIP-seq analysis of acute lymphoblastic leukemia (ALL) cell lines 697 and Nalm6 treated with prednisolone (10 μM for 697 and 5 μM for Nalm6, 24 h) [53], was selected for further analysis. Raw data were downloaded from GEO using the SRA Toolkit [54]. Read quality was assessed with FastQC v0.11.9 and MultiQC v1.14 [55,56], trimmed using Trimmomatic v0.39 [57], and aligned to the hg38 human genome with Bowtie2 [58]. SAMtools v1.22 [59] was used for file conversion and indexing. Peak calling was performed with HOMER v4.11 and MACS2 [60,61]; motif enrichment and annotation were also done with MEME-Suite tool [62]. Peak visualization was carried out using ChIPseeker and the UCSC Genome Browser [63,64,65]. The UCSC session is available at: https://genome.ucsc.edu/s/pochoa/ALL_hg38_HSPB1 (accessed on 29 April 2025).

### 2.7. Bioinformatics

Publicly available RNA-seq datasets were obtained for the ENZ-resistant VCaP cell line (GSE179157) and from a previous analysis of DTX-resistant PCa cells from our group [66,67], and used for subsequent analysis. In a previous study, VCaP cells were implanted into male nude mice that were castrated to induce castration-resistant tumors, leading to the development of castration-resistant tumors from which VCaP-CRPC cells were derived [66]. Following ENZ treatment until tumor recurrence, these VCaP-ENZR cells were isolated, maintained in ENZ-supplemented medium, and subjected to RNA sequencing [66]. Differentially Expressed Genes (DEGs) were identified using the following thresholds: q-value < 0.05 and log fold change > 1. Heatmap was generated using GraphPad Prism, version 8.2.1. Pathway enrichment analysis was conducted using Enrichr [68], accepting enriched pathways with q-value < 0.05.

### 2.8. Kaplan–Meier Survival Curves

Clinical data from PCa patients were derived from real-world insurance claims. Overall survival (OS) was defined as the time from tissue collection to the patient’s last recorded clinical activity. Kaplan–Meier (KM) survival curves were generated for groups stratified by molecular features and treatment regimens. Gene expression groups were defined using a three-gene panel (*NR3C1*/GR, *PSIP1*/LEDGFp75, and *HSPB1*/HSP27). For each gene, transcript per million (TPM) values were dichotomized at the median (50th percentile). Tumor samples were classified as high expression if all three genes were expressed above their respective medians, and as low expression if all three genes were expressed below their respective medians. Samples with mixed expression patterns were excluded from the high–low comparison. These stratified groups were subsequently used to generate KM survival curves for overall survival (OS). Furthermore, curves corresponding to overall tumors included those for all specimen sites (primary + metastatic), primary tumors included the prostate as the only specimen site, and metastatic tumors included only other non-prostate specimen sites. Prostate tumors of unknown specimen sites were excluded from KM survival curves. Patient demographic information was included in Supplementary Table S3. Note that the demographics table did not exactly match the numbers in the KM curves because tumors that did not have a last contact date were excluded from the curves. The KM curves are reflective of overall survival regardless of treatment. Hazard ratios (HRs) were calculated using the Cox proportional hazards model, and survival differences between groups were evaluated using the log-rank test. Statistical significance was determined at *p* < 0.05.

### 2.9. Statistics

Statistical analysis and graph generation were performed using GraphPad Prism, version 8.2.1. Unpaired *t* test was used to analyze differences between treatment groups and mean ± standard error of the mean (SEM) was calculated from a minimum of 3 independent experiments and statistical significance was determined at *p* < 0.05.

## 3. Results

### 3.1. Enzalutamide Resistance Confers Resistance to Docetaxel in PCa Cells

**Figure 1:**
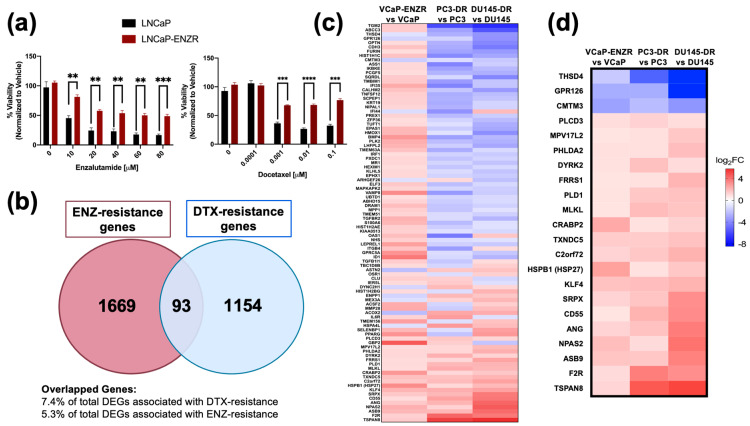
ENZ resistance confers cross-resistance to DTX and exhibits shared gene signatures with DTX resistance in PCa cells. LNCaP and LNCaP-ENZR cells were treated with increasing concentrations of ENZ and DTX (**a**). Cell viability was evaluated using MTT assays following 72 h of drug treatment, with DMSO as vehicle control. Statistical analysis was performed using unpaired *t* test. ** *p* < 0.01, *** *p* < 0.001, **** *p* < 0.0001. Error bars represent mean ± SEM from 3 independent experiments. (**b**) Venn diagram of differentially expressed genes (DEGs) associated with both ENZ-resistance and DTX-resistance in PCa cell lines revealed 93 overlapping genes. (**c**) Heatmap of the 93 overlapping genes associated with ENZ/DTX cross resistance. (**d**) Heatmap of 22 overlapping genes exhibiting a shared expression pattern associated with ENZ/DTX cross-resistance. The red box highlights *HSPB1*, the gene encoding HSP27.

The development of resistance to ENZ in PCa cells can be associated with cross-resistance to subsequent treatments with ARSI or taxane drugs [69]. To gain insights into mechanisms underlying this drug cross-resistance we first evaluated the cytotoxic effects of ENZ and DTX on LNCaP and LNCaP-ENZR PCa cells. Our results revealed that LNCaP-ENZR cells displayed higher viability than their drug-sensitive counterparts when exposed to increasing concentrations of ENZ up to 80 μM (Figure 1a). These cells also exhibited resistance up to 0.1 μM DTX (Figure 1a), a concentration that is 10 times higher than the concentration of DTX (10 nM) used to maintain DTX-resistant PCa cells in culture.

LNCaP-ENZR cells also displayed resistance to increasing concentrations of the ARSI drug abiraterone acetate (Supplementary Figure S1a), known to exert cytotoxic effects on LNCaP cells via direct binding to AR [70], as well as resistance to the taxane CBZ (Supplementary Figure S1b). We also treated DTX-sensitive and -resistant PC3, DU145 and 22Rv1 PCa cells with increasing concentration of DTX or ENZ. While all the DTX-resistant cells showed resistance to DTX compared to their sensitive counterparts, we observed no significant differences in responses to ENZ between DTX-sensitive and DTX-resistant cells for all three pairs (Supplementary Figure S2). This may be explained by the presence of GR in all these cell lines, as well as ARv7 in 22Rv1 cells [28]. High ENZ concentrations (>40 μM) caused moderate cytotoxicity in all cell lines except PC3-DR.

### 3.2. HSP27 Is Upregulated in ENZ/DTX Cross-Resistant PCa Cells

Next, we analyzed publicly available RNA-seq data comparing DTX-resistant [67] PC3-DR and DU145-DR cells and ENZ-resistant VCaP cells (GSE179157) to their sensitive counterparts to identify common gene signatures. Using a cutoff value of *p* < 0.05 and log_2_FC < 1, we identified 93 overlapping differentially expressed genes (DEGs) between the two drug resistant groups (Figure 1b). A heatmap was generated to examine the gene expression profiles among the 93 common genes. Gene expression was compared across VCaP-ENZR vs. VCaP, PC3-DR vs. PC3, and DU145-DR vs. DU145, which further revealed common gene signatures between the ENZ-resistant and DTX-resistant cell lines (Figure 1c). Among the DEGs, 19 were upregulated and 3 downregulated in both resistant groups (Figure 1d). Gene pathway analysis for the 93 DEGs revealed several enriched cancer-related pathways including p53 pathway, hypoxia, apoptosis, and negative regulation of programmed cell death, among others (Supplementary Figure S3).

To validate the RNA-seq results, we focused on genes associated with therapy resistance, particularly those involved in apoptotic processes. The gene *HSPB1* drew our attention as it was associated with both apoptosis and cellular response to growth factor stimulus (Supplementary Tables S1 and S2). *HSPB1* encodes HSP27, a chaperone protein that antagonizes the mitochondrial-dependent apoptotic pathway by interacting with Bax and cytochrome c to prevent caspase activation [42,43,71]. Since HSP27 is a known driver of therapy resistance in PCa and other cancers [43,44,45,46,47,48] and is regulated by the PCa chemoresistance-associated protein LEDGF/p75 [31,35,36], a GR target, we focused subsequent studies on examining the contribution of the GR-LEDGF/p75-HSP27 axis to resistance to ENZ and DTX in PCa cells.

**Figure 2:**
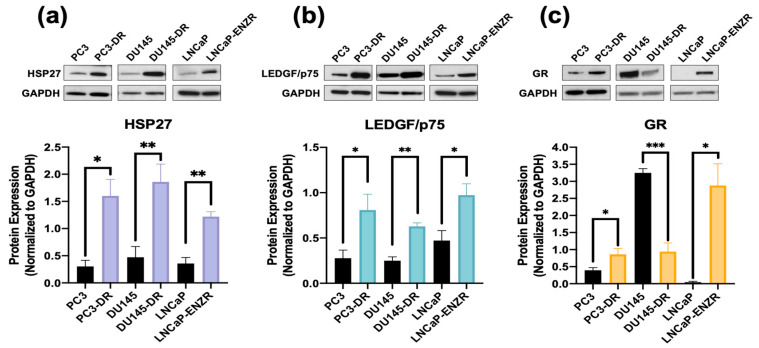
HSP27 upregulation in ENZ- and DTX-resistant PCa cells correlates with overexpression of GR and LEDGF/p75. Representative immunoblots and their quantification of the protein expression of (**a**) HSP27 and (**b**) LEDGF/p75, and (**c**) GR protein in PC3, PC3-DR, DU145, DU145-DR, LNCaP and LNCaP-ENZR cells. Quantified band values were obtained with ImageJ software and plotted as relative protein expression normalized to GAPDH. Statistics were performed using unpaired *t* tests comparing sensitive cell lines to their resistant counterparts. Error bars represent mean ± SEM from at least 4 independent blots for each cell line. * *p* < 0.05, ** *p* < 0.01, *** *p* < 0.001. GAPDH bands for LNCaP and LNCaP-ENZR in panels (**a**) and (**b**) originated from the same blot.

Immunoblotting revealed that HSP27 expression was significantly upregulated in PC3-DR, DU145-DR and LNCaP-ENZR cells compared to their parental, drug-sensitive counterparts (Figure 2a). In addition, using the same panel of drug-sensitive and -resistant PCa cell lines we confirmed the expression of LEDGF/p75, a known regulator of HSP27 in PCa cells [36,41]. Similarly to HSP27, LEDGF/p75 and GR expressions were enhanced in drug-resistant cells (Figure 2b,c). Prolonged ARSI treatment in PCa cells leads to upregulated GR expression, a feature associated with ARSI-resistance [15,16], and this was observed in our LNCaP-ENZR cells (Figure 2c). Previously, we demonstrated that GR regulates and interacts with LEDGF/p75 to form a transcriptional complex that promotes taxane resistance in PCa [31]. Additionally, while GR protein expression levels are downregulated in DU145-DR cells (Figure 2c), we reported a rapid increase in translocated nuclear GR (activated GR) in these cells in the presence of glucocorticoids [28]. HSP27 and GR expression levels were also upregulated in the AR+/GR+ 22Rv1-DR PCa cell line compared to their sensitive counterpart. However, there was no difference in LEDGF/p75 expression, most likely due to the combined effects of AR and GR signaling, both of which induce LEDGF/p75 expression [29,31] (Supplementary Figure S4).

### 3.3. GR and LEDGF/p75 Influence HSP27 Expression

**Figure 3:**
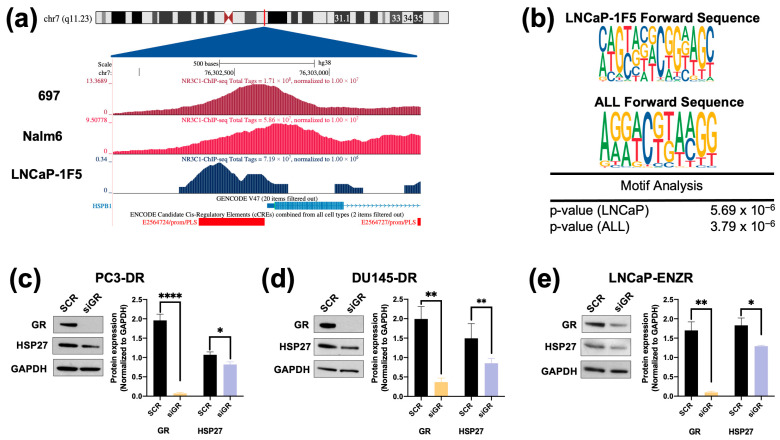
ChIP-seq analysis identifies GR occupancy at the HSP27 promoter, and silencing GR leads to cell line-specific decrease in HSP27 protein expression levels in DTX- and ENZ-resistant PCa cells. Chromatin immunoprecipitation sequencing (ChIP-seq) for the glucocorticoid receptor (GR/NR3C1) was performed in prostate cancer and acute myeloid leukemia cell lines using datasets GSE30623 and GSE175482. (**a**) Visualization using the UCSC Human Genome Browser (GRCh38) shows normalized *NR3C1* binding peaks near the transcription start site (TSS) of *HSPB1,* the gene encoding HSP27. ChIP-seq tracks are depicted in shades of blue, the GENCODE v43 *HSPB1* transcript is shown in blue, and ENCODE promoter-like elements are highlighted in red. (**b**) Motif analysis using the MEME Suite tool identified potential *NR3C1* binding sites in LNCaP-1F5, 697, and Nalm6 cell lines. DTX-resistant PCa cell lines PC3-DR (**c**), DU145-DR (**d**), and ENZ-resistant LNCaP-ENZR (**e**) were transfected with siRNA specific for GR or scrambled negative control oligos (SCR) for 72 h, leading to significant HSP27 downregulation. Quantified band values for GR and HSP27 were obtained with ImageJ software and plotted as relative protein expression normalized to GAPDH. Statistical analyses were performed using unpaired *t* tests comparing SCR to siLEDGF or siGR samples. * *p* < 0.05, ** *p* < 0.01, **** *p* < 0.0001. Error bars represent mean ± SEM from at least 3 independent experiments for each cell line.

While LEDGF/p75 has been shown to bind to the promoter region of *HSPB1* [36,38], there is currently no evidence of GR binding to this region. Preliminary screening of publicly available ChIP-seq datasets for potential GR binding sites within this promoter region revealed several studies reporting GR binding to this region. For further analysis, we selected ChIP-seq datasets from independent studies focused on the glucocorticoid-treated PCa cell line LNCaP-1F5, which was engineered to overexpress GR [51,52]. In addition, the acute lymphoblastic leukemia (ALL) cell lines 697 and Nalm6, which endogenously overexpress GR [53], were also included to assess GR binding at HSP27 promoter or enhancer regions, following the workflow we described previously [31]. Representative peaks indicating GR binding at the *HSPB1* gene promoter region in LNCaP-1F5 and the two ALL cell lines are shown in Figure 3a. GR binding motifs near the *HSPB1* transcriptional start site were also identified in the three cell lines (Figure 3b).

To determine whether increased HSP27 expression in the DTX-resistant and ENZ-resistant PCa cell lines was dependent on LEDGF/p75 expression, we knocked down the latter in these cell lines. HSP27 expression was significantly reduced in DU145-DR and LNCaP-ENZR cells, but not in PC3-DR cells, upon LEDGF/p75 depletion (Supplementary Figure S5a–c). Given that our group previously identified LEDGF/p75 as a GR-regulated protein involved in DTX resistance in PCa cells [31], we next sought to evaluate whether HSP27 protein expression is also dependent on GR. HSP27 expression was significantly decreased upon GR silencing in all drug-resistant PCa cell lines (Figure 3c–e).

### 3.4. Targeting HSP27 Re-Sensitizes DTX-Resistant PCa Cells to DTX

**Figure 4:**
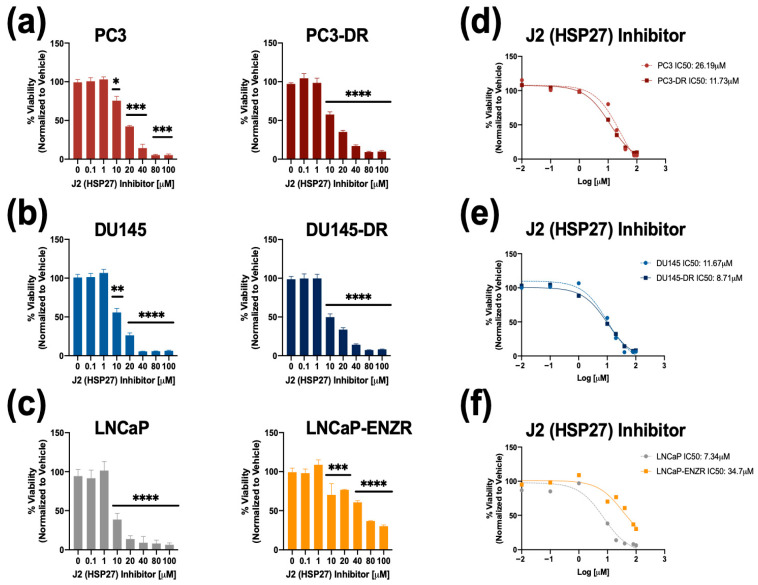
The HSP27 inhibitor J2 reduces cell viability in drug-sensitive and -resistant PCa cells. Cell viability was assessed using MTT assays after drug treatments for 72 h. DMSO was used as vehicle control. J2 dose response was performed in (**a**) PC3 and PC3-DR, (**b**) DU145 and DU145-DR, (**c**) LNCaP and LNCaP-ENZR. IC50 values were calculated for J2 in (**d**) PC3 and PC3-DR, (**e**) DU145 and DU145-DR, and (**f**) LNCaP and LNCaP-ENZR cells. Statistical analyses were performed using unpaired *t* tests. * *p* < 0.05, ** *p* < 0.01, *** *p* < 0.001, **** *p* < 0.0001. Error bars represent mean ± SEM from at least 3 independent experiments for each cell line.

To determine the significance of HSP27 blockade in ENZ-DTX cross-resistance, we targeted HSP27 using a commercially available inhibitor (J2) that induces abnormal dimer formation and attenuates the formation of HSP27 polymers to stimulate its degradation [72]. Recent studies showed that combining J2 with chemotherapeutic agents such as taxol, cisplatin, or 17-AAG, increased the response of chemoresistant lung adenocarcinoma to these agents [72]. To assess the cytotoxic effects of J2 in our PCa cell line panel, we treated both drug-sensitive and -resistant PCa cell lines with increasing concentrations of J2 inhibitor (0.1–100 µM) for 72 h. All PCa cell lines exhibited a significant decrease in cell viability at concentrations of 10 µM or above (Figure 4a–c). The calculated IC50 values of J2 were: 26.19 µM (PC3), 11.73 µM (PC3-DR), 11.67 µM (DU145), 8.71 µM (DU145-DR), 7.34 µM (LNCaP), and 34.7 µM (LNCaP-ENZR) (Figure 4d–f).

**Figure 5:**
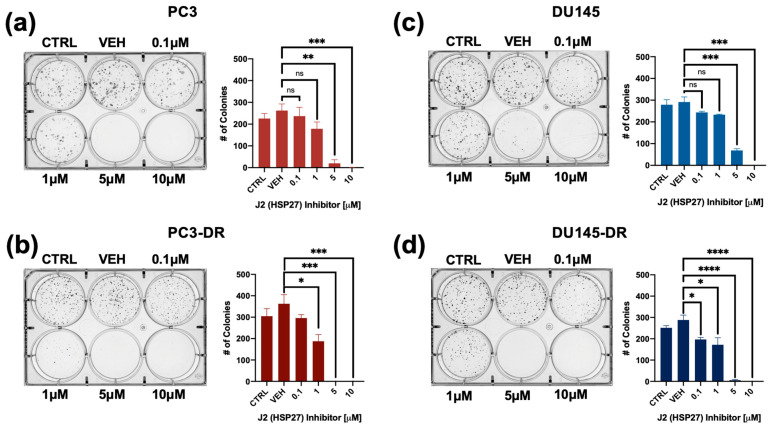
The HSP27 inhibitor J2 reduces colony formation in both DTX-sensitive and -resistant PCa cell lines. Representative images of colonies from (**a**) PC3, (**b**) PC3-DR, (**c**) DU145, and (**d**) DU145-DR cells show decreased clonogenic capacity for the four cell lines following treatment with J2. Bar graphs depict quantitative analysis of colony numbers. J2-treated groups were compared to their respective vehicle (VEH) controls. Colonies were assessed after 10 days. Statistical significance was determined using unpaired *t*-tests. * *p* < 0.05, ** *p* < 0.01, *** *p* < 0.001, **** *p* < 0.0001. ns = not significant. Error bars represent mean ± SEM from at least 3 independent experiments for each cell line.

To further evaluate the inhibitory potential of J2 in DTX-resistant cells we conducted colony formation assays. It was not possible to include LNCaP and LNCaP-ENZR in this experiment due to their inability to form clearly distinct colonies. Notably, PC3-DR and DU145-DR cells, but not their DTX-sensitive counterparts exhibited a significant reduction in colony formation at 1 µM J2 (Figure 5a–d) compared to vehicle control. While this concentration did not significantly affect cell viability, as assessed by MTT assays (Figure 4), it led to a greater impairment of clonogenic potential in the DTX-resistant cell lines.

**Figure 6:**
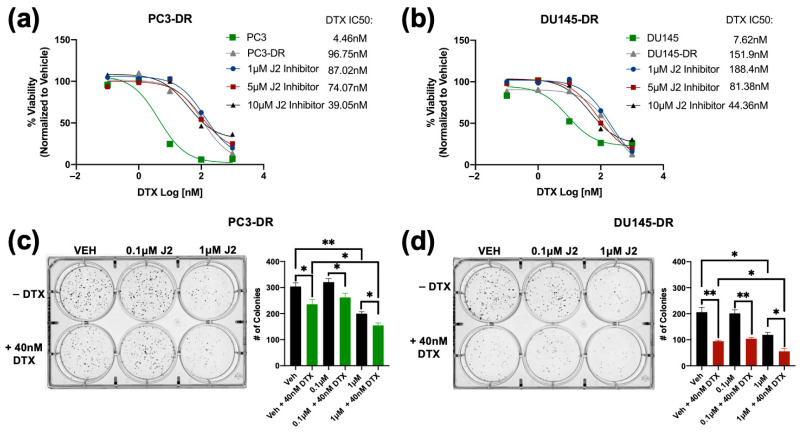
The HSP27 inhibitor J2 re-sensitizes chemoresistant PCa cells to DTX. Cell viability was evaluated by MTT assays after 72 h of treatment, with DMSO used as vehicle control. DTX (0.1 nM, 1 nM, 10 nM, 100 nM, and 1000 nM) dose response assays were conducted in PC3-DR and DU145-DR cells in the presence of 1 µM, 5 µM, or 10 µM J2 (**a**,**b**). Representative images of colony formation assays show reduced clonogenic potential in PC3-DR and DU145-DR cells treated with J2 (0.1 µM or 1 µM) plus DTX (40 nM) compared to vehicle or J2 alone (**c**,**d**). Bar graphs provide quantitative analysis of colony numbers, with comparisons made to respective VEH controls. Data represent at least three independent experiments per cell line. * *p* < 0.05, ** *p* < 0.01,. Error bars indicate mean ± SEM from at least 3 independent experiments.

Next, we used MTT assays to assess the combinatorial effects of J2 plus DTX on the resistant cells by treating PC3-DR and DU145-DR with 1 μM, 5 μM and 10 μM of J2 in combination with increasing concentrations of DTX (0.1 nM, 1 nM, 10 nM, 100 nM, and 1000 nM). The J2 concentrations were selected based on the results from Figure 4 indicating IC50 values around 10 μM in these DR cell lines. IC50 values for DTX were also calculated for PC3 (4.46 nM), PC3-DR (96.75 nM), DU145 (7.62 nM) and DU145-DR (151.9 nM) to determine changes in cellular responses to DTX in combination with J2. The combinatorial treatment of J2 (1 μM, 5 μM and 10 μM) plus DTX (0.1 nM, 1 nM, 10 nM, 100 nM, and 1000 nM) increased DTX cytotoxicity in PC3-DR cells as the DTX IC50 values decreased to 87.02 nM, 74.07 nM and 39.05 nM, respectively (Figure 6a). Similarly, the DU145-DR cells exhibited reduced IC50 values when J2 was combined with DTX (Figure 6b).

We then evaluated the effect of targeting HSP27 in combination with DTX on clonogenic formation. For this experiment we treated PC3-DR and DU145-DR cells with 0.1 μM or 1 μM of J2 in the presence and absence of 40 nM DTX. In the absence of DTX, treatment with 0.1 μM J2 did not affect clonogenic capacity; however, there was significant reduction at 1 μM J2 in both PC3-DR and DU145-DR cell lines (Figure 6c,d). Notably, combined treatment with 40 nM DTX and 1 μM J2 resulted in further reduction in colony formation compared to DTX alone or J2 alone (Figure 6c,d), suggesting that targeting HSP27 in combination with DTX improves the response to taxanes in chemoresistant PCa cells.

### 3.5. Targeting HSP27 in Combination with ENZ or DTX Reduces Tumorsphere Formation in Drug-Resistant PCa Cells

**Figure 7:**
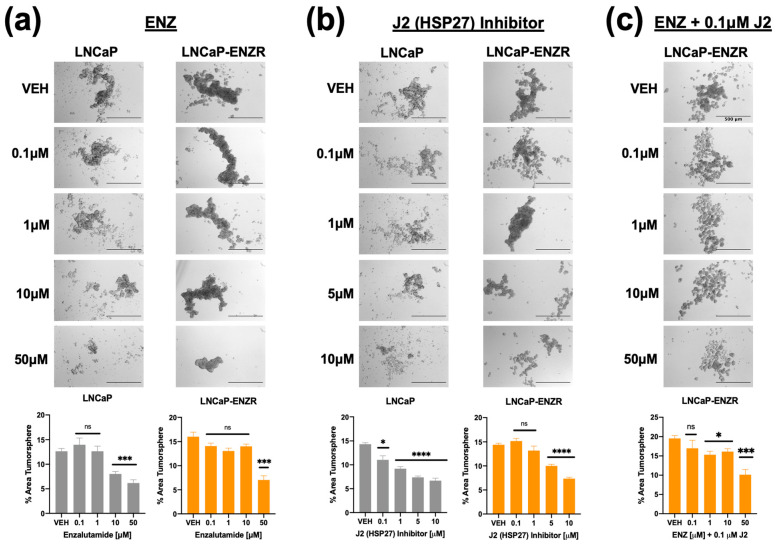
Inhibition of HSP27 with J2 in combination with ENZ reduces tumorsphere formation in both ENZ- sensitive and ENZ-resistant PCa cells. LNCaP and LNCaP-ENZR cells were cultured in MammoCult media supplemented with various concentrations of ENZ alone (0.1 μM, 1 μM, 10 μM, 50 μM) (**a**), J2 alone (0.1 μM, 1 μM, 5 μM, 10 μM) (**b**), or a combination of J2 (0.1 μM) with ENZ (0.1 μM, 1 μM, 10 μM, 50 μM) (**c**) for five days. DMSO was used as the vehicle control. Images were captured using an Olympus IX70 microscope at 4× magnification, with a scale bar of 500 μm applied to all representative images. Total tumorsphere area was quantified from four images per condition for each cell line using Image J. Data are presented as the mean ± SEM from at least 3 independent experiments. Statistical significance is indicated as follows: * *p* < 0.05, *** *p* < 0.001, **** *p* < 0.0001. ns = not significant.

Previously, we showed that tumorsphere formation in DTX-resistant PCa cells can be attenuated by targeting LEDGF/p75 [30]. To assess the impact of targeting HSP27 on tumorsphere formation in drug-resistant PCa cells, we treated LNCaP and LNCaP-ENZR cells with varying concentrations of ENZ alone (0.1 μM, 1 μM, 10 μM, 50 μM), J2 alone (0.1 μM, 1 μM, 5 μM, 10 μM), or a combination of J2 (0.1 μM) with increasing ENZ doses. ENZ monotherapy significantly reduced tumorsphere formation at 10 μM in LNCaP cells, while the LNCaP-ENZR cells only responded partially at 50 μM (Figure 7a). Similarly, J2 alone decreased tumorsphere formation in LNCaP cells at 0.1 μM, but LNCaP-ENZR cells required 5 μM for a comparable effect (Figure 7b). This suggested that LNCAP-ENZR tumorspheres were more resistant to both ENZ and the J2 inhibitor. We then examined whether co-treatment with J2 (0.1 μM—a non-cytotoxic dose alone as shown in Figure 4c) and increased ENZ concentrations could sensitize LNCaP-ENZR cells to ENZ. This combination significantly enhanced cellular response to ENZ with reduced tumorsphere formation observed as low as 1 μM ENZ + 0.1 μM J2 (Figure 7c). We also assessed the effect of DTX alone and in combination with J2 in LNCaP and LNCaP-ENZR cells. Both cell lines showed reduced spheroid formation starting at 1 nM DTX (Supplementary Figure S4a). The combination of DTX with J2 had a similar effect (Supplementary Figure S4b).

**Figure 8:**
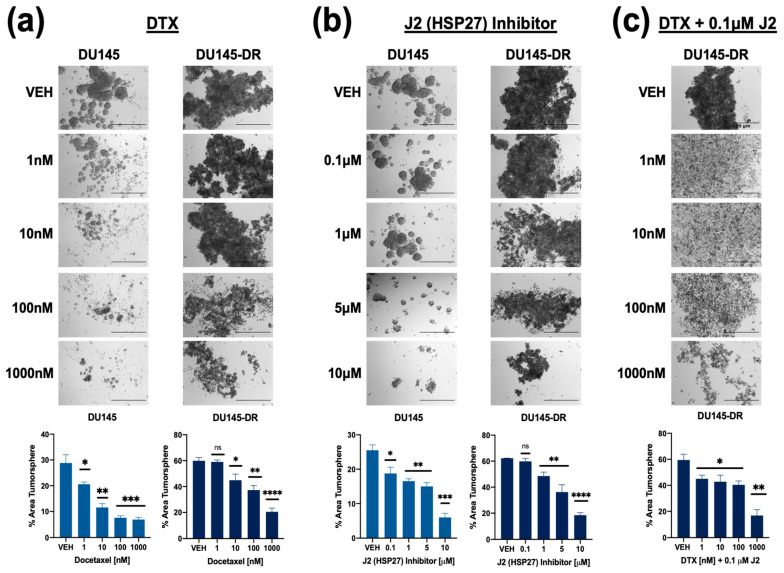
Combining HSP27 inhibition with DTX treatment reduces tumorsphere formation in both DTX-sensitive and -resistant PCa cells. DU145 and DU145-DR cells were cultured in MammoCult media supplemented with varying concentrations of DTX (1 nM, 10 nM, 100 nM, 1 μM) (**a**), J2 (0.1 μM, 1 μM, 5 μM, 10 μM) (**b**), or a combination of J2 (0.1 μM) plus DTX (1 nM, 10 nM, 100 nM, 1 μM) (**c**) for five days. DMSO was used as a vehicle control. Images were captured using an Olympus IX70 microscope at 4× magnification, with a scale bar of 500 μm applied to all representative images. Total tumorsphere area was quantified from four images per condition for each cell line using Image J. Data represent the mean ± SEM from at least 3 independent experiments. Statistical significance was determined as follows: * *p* < 0.05, ** *p* < 0.01, *** *p* < 0.001, **** *p* < 0.0001. ns = not significant.

A similar experiment was conducted with DU145 and DU145-DR cell lines. Cells were treated with DTX alone (1 nM, 10 nM, 100 nM, 1000 nM), J2 alone (0.1 μM, 1 μM, 5 μM, 10 μM), or in combination. In DU145 cells, DTX alone reduced tumorsphere formation starting at 1 nM, while, as expected, DU145-DR cells required at least 10 nM for a significant effect (Figure 8a). J2 alone also decreased tumorsphere formation in DU145 cells at 0.1 μM, whereas DU145-DR cells responded at 1 μM and above (Figure 8b). However, combining 0.1 μM J2 with DTX led to significant tumorsphere reduction in DU145-DR cells at 1 nM DTX (Figure 8c).

### 3.6. High Tumor Expression of the GR-LEDGF/p75-HSP27 Axis Is Associated with Decreased Overall Survival in PCa Patients

**Figure 9:**
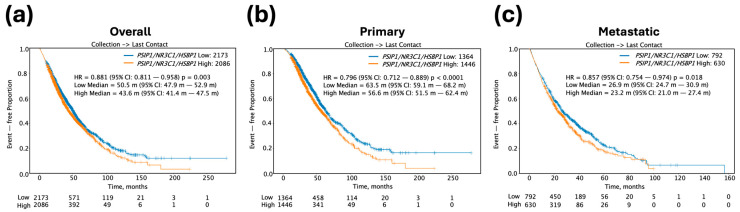
Kaplan–Meier survival curves for PCa patient cohorts stratified by high versus low transcript expression of the target three-gene panel including *PSIP1* (LEDGFp75), *NR3C1* (GR), and *HSPB1* (HSP27). (**a**) OS for all PCa patient tumor samples (n = 4259), including primary and metastatic tumors, comparing patients with low (blue curve) versus high (orange curve) transcript expression of the three-gene panel. (**b**) OS for primary PCa patient tumor samples (n = 2810) comparing low versus high gene panel expression. (**c**) OS for metastatic PCa patient tumor samples (n = 1422) comparing low versus high gene panel expression.

The results shown above implicated the GR-LEDGFp75-HSP27 axis in PCa resistance to both ENZ and DTX. Therefore, we predicted that high expression of this gene panel in prostate tumors may negatively influence overall survival (OS) in PCa patients. To investigate the prognostic significance of the tumor expression levels of our target gene panel—*NR3C1* (encodes GR), *PSIP1* (encodes LEDGF/p75), and *HSPB1* (encodes HSP27)—in PCa patients, we analyzed OS patient data from the Caris Life Sciences clinico-genomic database, calculated from the time of tissue collection to the time of last contact with the patient. In a full PCa patient cohort (n = 4259), patients with high tumor expression of the three genes (Median = 43.6 months [m]; confidence interval [CI]: 41.4–47.5 m) exhibited a significantly shorter median OS by 6.9 m (hazard ratio [HR] = 0.881; 95% confidence interval [CI]: 0.811–0.958; *p* = 0.003) when compared to patients with low tumor expression (Median = 50.5 m; 95% CI: 47.9–52.9 m) (Figure 9a). Further analysis of primary tumor samples (n = 2810) revealed that high tumor expression of the three-gene panel was associated with an equal reduction in median OS by 6.9 m (HR = 0.796; 95% CI: 0.712–0.889; *p* < 0.0001) when compared to low tumor expression (Figure 9b). Likewise, in 1422 patients with metastatic tumor samples, high tumor expression of the three-gene panel was associated with a median OS decrease of 3.7 m (HR = 0.857; 95% CI: 0.754–0.974; *p* < 0.018) when compared to low tumor expression (Figure 9c). These findings underscore the potential of the *NR3C1-PSIP1-HSPB1* gene expression panel as a prognostic biomarker for OS in PCa patients with primary and metastatic disease.

## 4. Discussion

The sequential treatment of PCa patients with ARSI and taxane drugs has been associated with acquisition of drug cross-resistance [26,69,73,74]. For instance, resistance to ENZ induces cross-resistance to other ARSI drugs and to DTX in mCRPC, both in vitro and in vivo, and to CBZ in vitro [69,74,75]. Similarly, DTX-resistant PCa cells display cross-resistance to other taxanes [49]. Most mechanistic studies on PCa drug cross-resistance have focused on the role of AR, particularly its splice variant AR-V7, which mediates resistance to ARSIs but not to taxanes in PCa cells and tissues [11,14,26,75,76,77]. Wnt/β-catenin signaling has also been identified as a potential cross-resistance mechanism, as β-catenin interacts with AR and GR and contributes to resistance to both ARSI and DTX [28,78,79,80,81].

GR upregulation and activation, known to drive cross-resistance between different ARSI drugs [15,16,17,18,19,20,21,22,23,82,83], have been observed in DTX-treated patients and DTX-resistant cellular models such as PC3-DR and DU145-DR [27,28,30,31]. Of note, these DR cell lines are considered as t-NEPC-like since they show elevated expression of chromogranin A and stemness markers [84]. Consistent with a previous study implicating GR in t-NEPC development [25], these cell lines express GR but lack AR expression [28,29,30,31]. Also consistent with observations [69], we observed that our LNCaP-ENZR cells showed robust cross-resistance to DTX, CBZ, and abiraterone. Of note, treatment of LNCaP-ENZR cells with taxanes did not appear to have dose-dependent effects beyond 1 nM for DTX or 10 nM for CBZ, which was indicative of cross-resistance to high taxane concentrations. By contrast, the parental LNCaP cells exhibited strong responses to 1 nM DTX (<30% viability) with similar effects beyond this concentration, and a more potent dose-dependent response was achieved with CBZ in these cells. We cannot rule out that the LNCaP cell cultures may have sub-populations that exhibit intrinsic resistance to DTX. Alternatively, drug-induced stress may lead to the activation of compensatory stress responses—such as mitochondrial overactivation—in a sub-population of LNCaP cells, which could paradoxically support short-term cell survival or increased dehydrogenase activity, as measured by the MTT assay, in the presence of high DTX concentrations.

The mechanisms by which GR promotes resistance between ARSI treatment and taxane chemotherapy are not well understood. However, targeting GR or its direct target gene Mono Amine Oxidase-A (MAO-A), a mitochondrial oxidoreductase, was shown to enhance the efficacy of both ARSI and DTX, possibly by reducing the expression of anti-apoptotic genes like Bcl-xL and Bcl-2 [27,85]. We also reported that LEDGF/p75, an epigenetic reader and transcription co-activator that promotes cancer chemoresistance [32,33,86], is a target gene of GR and part of a large oncogenic transcription complex in active chromatin of DTX-resistant PCa cells that includes GR, β-catenin, c-MYC, and Menin [30,31]. Upregulation of GR in LNCaP-ENZR cells correlated with upregulation of LEDGF/p75, and GR silencing in these cells and DTX-resistant PCa cells led to significant LEDGF/p75 downregulation [31]. In addition, pharmacological targeting of GR in DTX-resistant cells with selective GR modulators (SGRM) increased their chemosensitivity by decreasing Bcl-2 levels and increasing apoptosis, whereas combined targeting of GR and LEDGF/p75 decreased the clonogenic capacity of these cells in the presence of DTX [31].

To gain additional mechanistic insights into the cross-resistance between ARSI and taxanes we conducted in the present study an RNA-seq analysis that identified 93 DEGs commonly altered in both ENZ- and DTX-resistant PCa cell lines. Pathway enrichment analysis revealed that these genes were significantly associated with several cancer-related pathways including p53 signaling, hypoxia, and apoptosis. Focusing on genes with consistent expression patterns across all ENZ- and DTX-resistant cell lines, we identified three downregulated genes—*THSD4*, *GPR126*, and *CMTM3*—and nineteen upregulated genes, including *TSPAN8*, *HSPB1*, *ASB9*, *F2R*, and *NPAS2*. CMTM3 functions as a tumor suppressor involved in cell–cell adhesion and is downregulated in PCa tissues compared to benign prostatic hyperplasia [87]. It has also emerged as a potential therapeutic target due to its role in modulating the tumor microenvironment and functioning as an immune checkpoint regulator [88]. Similarly, THSD4, a tumor microenvironment modulator, is downregulated in PCa and in cisplatin-resistant lung cancer cells, suggesting a potential link to therapy resistance [89,90]. Among the upregulated genes, TSPAN8 was one of the most prominent. A member of the tetraspanin family of membrane glycoproteins, TSPAN8 is upregulated in several cancers including pancreatic, colorectal, gastric, liver, lung, breast, and ovarian cancers [91]. It has been associated with cancer stemness, implicated in drug resistance in breast cancer [92], and shown to promote invasion, migration, and autophagy in colorectal cancer [93]. Similarly, NPAS2, a circadian rhythm gene, is upregulated in gastric, breast, lung adenocarcinoma, and PCa [94,95,96,97], and contributes to chemoresistance through its role in DNA repair via binding to H2AX mRNA [98]. F2R, also known as protease-activated receptor 1 (PAR-1), is involved in thrombin-mediated signaling and is elevated in multiple cancers, where it promotes angiogenesis and tumor proliferation [99,100,101,102,103,104,105]. ASB9 is a relatively understudied E3 ligase identified as a cancer-related gene with prognostic value in various cancer types [106,107]. The mechanistic contribution of these genes to ENZ-DTX cross-resistance in PCa cells remains to be explored.

We focused our study on *HSPB1* since it is a well-established target of LEDGF/p75 that has been implicated in cancer chemoresistance [35,36,37,38,39,43]. Elevated expression of HSP27 has been linked to tumorigenesis, metastasis, and invasiveness in PCa and several cancer types [43,44,71,72,108]. Our results provided evidence supporting GR- and LEDGF/p75-mediated regulation of HSP27 in the context of drug cross-resistance by showing that (1) GR, LEDGF/p75, and HSP27 are upregulated in ENZ-resistant and DTX-resistant cell lines (with the exception of GR in DU145-DR cells as discussed in the Section 3); (2) GR or LEDGF/p75 silencing reduced HSP27 expression in ENZ-resistant and DTX-resistant PCa cells; and (3) ChIP-seq analysis from public datasets revealed GR enrichment at the *HSPB1* promoter in the LNCaP-1F5 PCa cell line and in two leukemia cell lines (696 and Nalm6), all of which express high levels of GR. Notably, LNCaP-1F5 and our LNCaP-ENZR cell line both overexpress GR [31,57,58], suggesting functional similarity and relevance to ENZ resistance mechanisms.

Our observation that LEDGF/p75 silencing significantly reduced the protein levels of HSP27 in DU145-DR cells is consistent with a report showing that HSP27 is transcriptionally regulated by LEDGF/p75 in DU145 PCa cells [41]. Previously, our group also reported that while the *HSP27* gene promoter is transcriptionally activated by LEDGF/p75 in PC3 cells, this activation is more robustly enhanced by overexpression of MeCP2, a LEDGF/p75 interacting partner [36]. This suggested that while LEDGF/p75 and GR may act as transcriptional regulators of HSP27, other transcription factors in the same transcriptional network may also be involved in this regulation. This could explain why robust silencing of LEDGF/p75 or GR in the ENZ- and DTX-resistant cell lines led to a significant but relatively modest decrease in HSP27 expression (Figure 3). Silencing LEDGF/p75 in DTX-resistant PCa models was shown in our previous studies to reduce cell viability, clonogenicity, and tumorsphere formation [30,49]. We also reported that pharmacological inhibition of GR combined with targeting of LEDGF/p75, or β-catenin, reduced cell viability, clonogenicity, and spheroid formation [28,31]. These observations supported their role in drug resistance and validated the mechanistic insights presented in this study.

The increased protein expression of the GR–LEDGF/p75–HSP27 axis in both ENZ-resistant and DTX-resistant PCa cell lines is consistent with a role in drug cross-resistance. Notably, ENZ has also been shown to induce apoptosis and downregulate anti-apoptotic genes, including HSP27, in PCa and other malignancies [109,110,111,112,113]. These findings underscore the therapeutic potential of targeting HSP27 as a strategy to mitigate drug cross-resistance in PCa. HSP27 has been implicated in resistance to multiple chemotherapeutic agents, including paclitaxel, temozolomide, 5-fluorouracil (5-FU), gemcitabine, and doxorubicin [43]. Inhibitors of HSP27, such as quercetin and RP101, have been shown to enhance the efficacy of anticancer therapies in several tumor models, including leukemia, glioblastoma, and oral cancers [108]. Consistent with these findings, our data show that treating ENZ- and DTX-resistant PCa cells with the HSP27 inhibitor J2, significantly increased their sensitivity to both drugs. This is consistent with recent studies showing that J2 sensitized lung adenocarcinoma cells to DTX [72].

Clinically, HSP27 inhibitors have been evaluated for their potential to enhance the efficacy of standard chemotherapy. For instance, OGX-427, an antisense oligonucleotide targeting HSP27, progressed to phase II clinical trials after demonstrating the ability to reduce circulating tumor cells and prostate-specific antigen (PSA) levels in PCa patients [114]. Another phase II trial “Borealis”, which enrolled 99 patients with metastatic bladder cancer, assessed survival benefit following treatment with a combination of OGX-427 and DTX compared to DTX alone [115]. However, this trial did not show significant survival benefit [115]. Similarly, other phase II trials evaluating OGX-427 in combination with carboplatin, gemcitabine, or paclitaxel in non-squamous cell carcinoma failed to demonstrate improved survival [116]. Although these trials did not show a survival benefit in patients treated with OGX-427, HSP27 is considered a candidate biomarker and therapeutic target given that its protein overexpression in various cancers including prostate tumors, determined by immunohistochemistry (IHC), is associated with poor prognosis [43,117]. While GR protein expression was found to be decreased, using IHC, in primary PCa it was increased in metastatic lesions [21]. Previous IHC analyses from our group showed that LEDGF/p75 protein expression is increased in PCa tissues compared to control tissues [118]. Although we did not perform IHC staining in this study, these protein-level findings from existing literature and databases align with our findings showing that overexpression of a 3-gene panel comprising *NR3C1* (GR)–*PSIP1* (LEDGF/p75)–*HSPBP1* (HSP27) in a large PCa patient cohort correlated with worse OS, with similar results among patients with primary and metastatic tumor samples profiled. We chose this 3-gene panel for our KM survival analysis over the individual genes in light of the growing reliance on limited gene panels or signatures as more informative prognostic PCa biomarkers compared to individual genes [119,120].

The GR–LEDGF/p75–HSP27 axis represents a promising therapeutic target to overcome drug cross-resistance in PCa. Our RNA-seq analysis further expands the repertoire of candidate genes and molecular pathways that may contribute to ENZ-DTX cross-resistance in PCa, supporting the growing body of evidence for a shared or overlapping genetic program, likely activated during treatment, underlying this cross-resistance. While we observed enhanced effects with combinatory treatments in functional assays, we acknowledge the limitations of not performing an in-depth quantitative analysis of drug combination effects in this study. Moreover, investigating the potential synergistic or additive effects of combining pharmacological inhibition of GR, LEDGF/p75, and HSP27 with different ARSI drugs and taxanes in preclinical models of mCRPC will be essential in future studies for advancing novel and more effective therapeutic strategies to the clinic.

## 5. Conclusions

This study highlights the role of the GR–LEDGF/p75–HSP27 axis in ENZ-DTX cross-resistance in PCa cells. It demonstrates that both GR and LEDGF/p75 influence HSP27 expression in ENZ- and DTX-resistant PCa cells, and that pharmacological targeting of HSP27 sensitizes resistant cells to ENZ and DTX. The study also underscores the value of a GR–LEDGF/p75–HSP27 gene expression panel in predicting OS in a large cohort of PCa patients. Future mechanistic studies are needed to explore the contribution of other DEGs identified in this study, as well as GR- and LEDGF/p75-target genes identified in our previous study [31], to ENZ-DTX cross-resistance. These studies would provide a strong foundation for dissecting the molecular basis of this cross-resistance, leading to the development of novel and effective therapeutic strategies for advanced PCa.

## Data Availability

The data presented here are available on request from the corresponding author. Publicly available RNA-seq data sets used in this study can be accessed through the following links: GSE179157, GSE30623, and GSE175482.

## Supplementary Materials

The following supporting information can be downloaded at https://www.mdpi.com/article/10.3390/cells14191566/s1, Figure S1: Enzalutamide resistance confers resistance to abiraterone and cabazitaxel in PCa cells; Figure S2: Docetaxel-resistant PCa cells show poor response to enzalutamide treatment. Figure S3: Enriched pathways associated with enzalutamide-docetaxel overlapping DEG set; Figure S4: HSP27 is upregulated in docetaxel-resistant 22Rv1 cells; Figure S5: Silencing LEDGF/p75 leads to cell line-specific decrease in HSP27 protein expression levels in docetaxel- and enzalutamide-resistant PCa cells. Figure S6: Inhibition of HSP27 in combination with docetaxel reduces tumorsphere formation in enzalutamide-resistant PCa cells; Table S1. Gene pathways found to be significant in MSigDB Hallmark 2020; Table S2. Gene pathways found to be significant in GO Biological Process 2025; Table S3. Patient Demographics.

## Abbreviations

The following abbreviations are used in this manuscript:

5-FU	5-fluorouracil
ADT	Androgen Deprivation Therapy
ALL	Acute Lymphoblastic Leukemia
AR	Androgen Receptor
ARSI	Androgen Receptor Signaling Inhibitor
AR-V7	Androgen Receptor Splice Variant 7
CBZ	Cabazitaxel
ChIP-seq	Chromatin Immunoprecipitation sequencing
CS-FBS	Charcoal-stripped Fetal Bovine Serum
DAXX	Death Domain-associated Protein
DEGs	Differentially Expressed Genes
DMSO	Dimethylsulfoxide
DTX	Docetaxel
DU145-DR	DTX-resistant DU145
ENZ	Enzalutamide
FBS	Fetal Bovine Serum
GAPDH	Glyceraldehyde-3-phosphate dehydrogenase
GR	Glucocorticoid Receptor
HR	Hazard Ratio
HRP	Horseradish Peroxidase
HSP27	Heat Shock Protein 27
IC50	Half-maximal Inhibitory Concentration
IHC	Immunohistochemistry
LEDGF/p75	Lens Epithelium-derived Growth Factor of 75 kDa
LNCaP-ENZR	ENZ-resistant LNCaP
MAO-A	Mono Amine Oxidase-A
mCRPC	Metastatic Castration-resistant Prostate Cancer
MTT	3-(4,5-Dimethylthiazol-2-yl)-2,5-Diphenyltetrazolium Bromide
OC2	ONECUT2
OS	Overall Survival
PC3-DR	DTX-resistant PC3
PCa	Prostate Cancer
PSMA-RLT	Prostate-Specific Membrane Antigen Radioligand Therapy
RNA-seq	RNA Sequencing
SCR	Scramble
SEM	Standard Error of the Mean
siRNA	Small Interfering RNA
SGRM	Selective GR Modulators
t-NEPC	Treatment-emergent Neuroendocrine PCa
VEH	Vehicle
