# Supplementary material for: The GR-LEDGF/p75-HSP27 Axis Contributes to Cross-Resistance Between Enzalutamide and Docetaxel in Prostate Cancer
Source: Cells. 2025 Oct 9;14(19):1566. doi: 10.3390/cells14191566 (PMC12523889; doi:10.3390/cells14191566)
Supplement: Supplementary file 1 [file cells-14-01566-s001.zip › cells-3823514-supplementary.pdf]

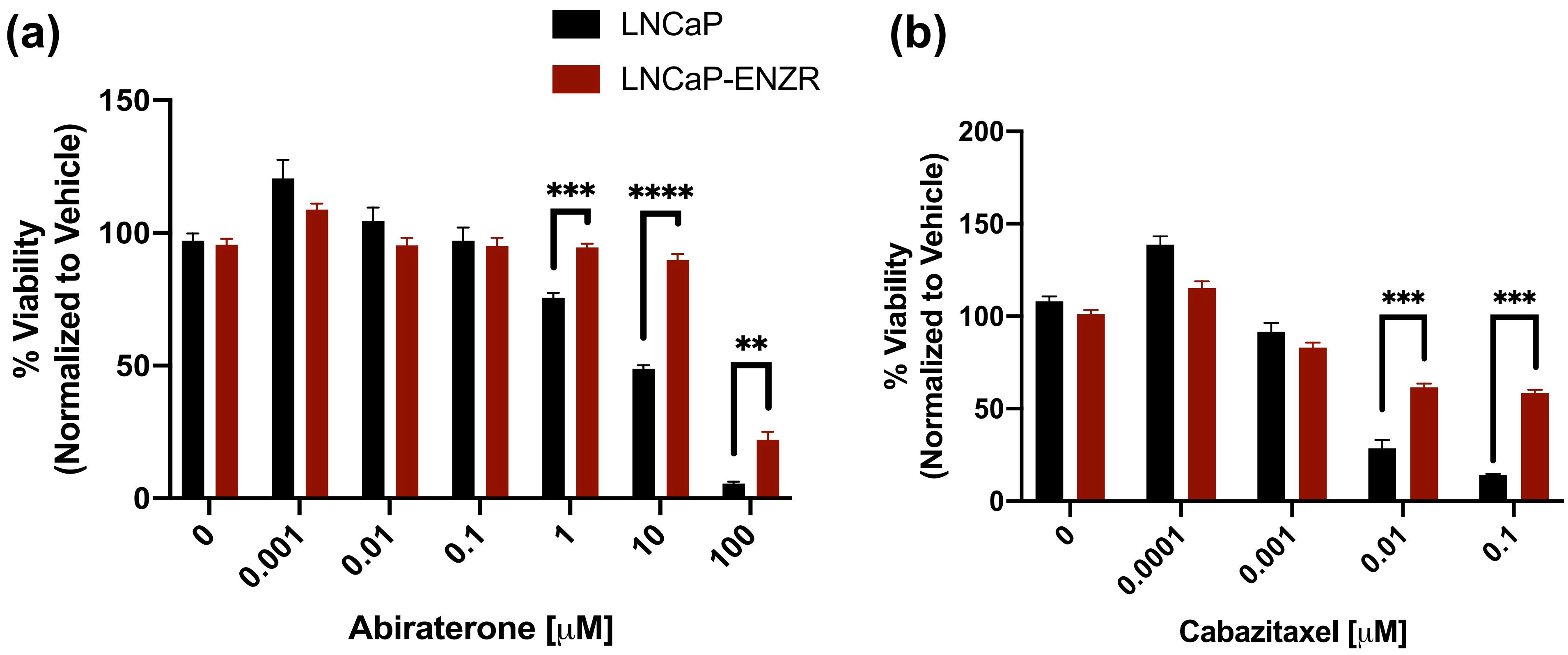

Figure S1. Enzalutamide resistance confers resistance to abiraterone and cabazitaxel in PCa cells. ENZ-sensitive LNCaP and ENZ-resistant LNCaP ENZ-R cells were treated with increasing concentration of (**a**) abiraterone (0.001 µM, 0.01 µM, 0.1 µM, 1 µM, 10 µM, and 100 µM) and (**b**) cabazitaxel (0.0001 µM, 0.001 µM, 0.01 µM, and 0.1 µM). Cell viability was evaluated using MTT assays following 72 hours of drug treatment, with DMSO serving as the vehicle control. Statistical analysis was performed using unpaired t test. *$p < 0.05$, **$p < 0.01$, ***$p < 0.001$, **** $p < 0.0001$. Error bars represent means +/- SEM from 3 independent experiments.

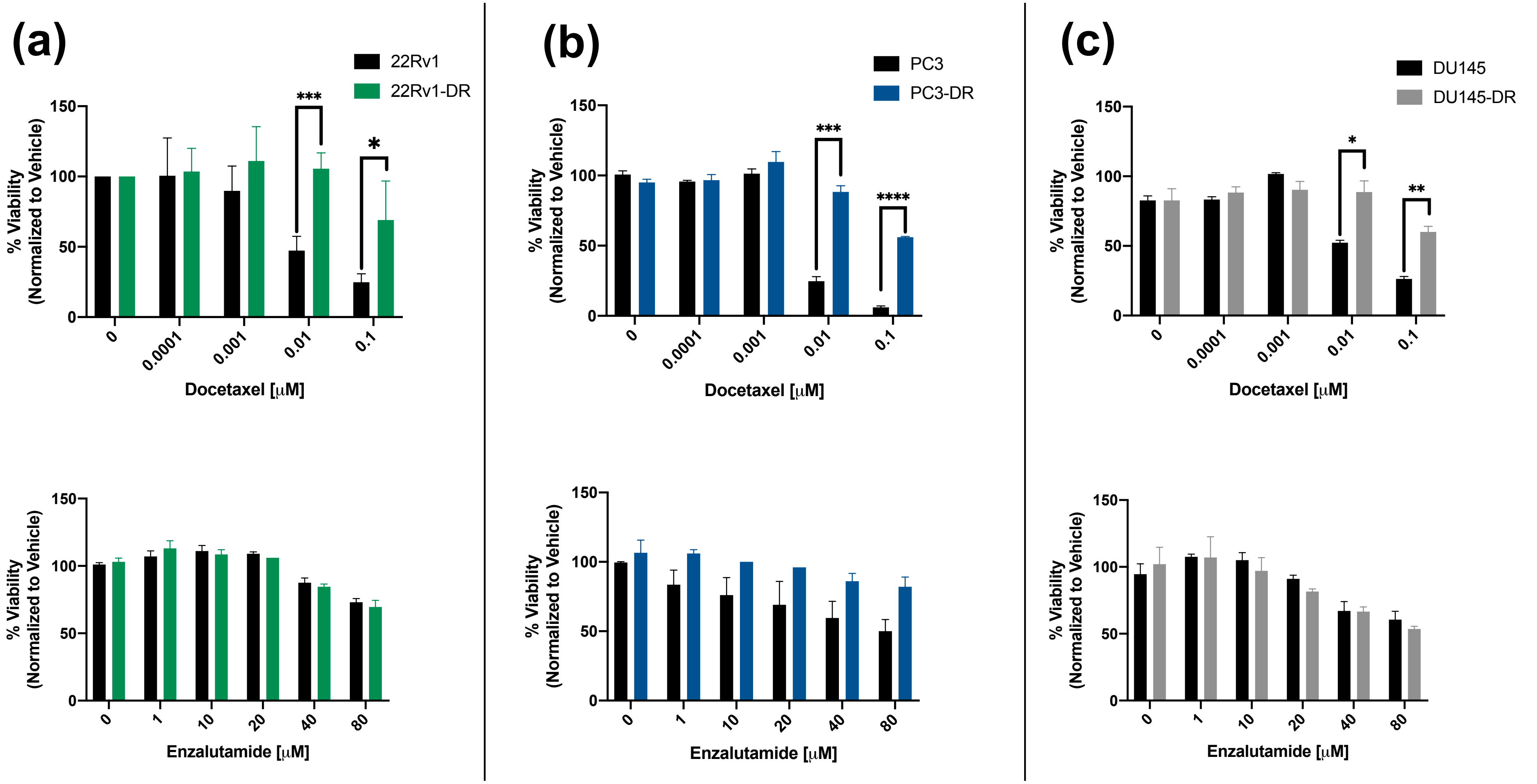

Figure S2. Docetaxel-resistant PCa cells show poor response to enzalutamide treatment. DTX-sensitive and –resistant (a) 22Rv1, (b) PC3, and (c) DU145 PCa cells were treated with docetaxel (0.0001 μM, 0.001 μM, 0.01 μM, and 0.1 μM) and enzalutamide (10 μM, 20 μM, 40 μM, 60 μM, and 80 μM). Cell viability was evaluated using MTT assays following 72 hours of drug treatment, with DMSO serving as the vehicle control. Statistical analysis was performed using unpaired t test. *p < 0.05, **p < 0.01, ***p < 0.001, **** p < 0.0001. Error bars represent means +/- SEM from 3 independent experiments.

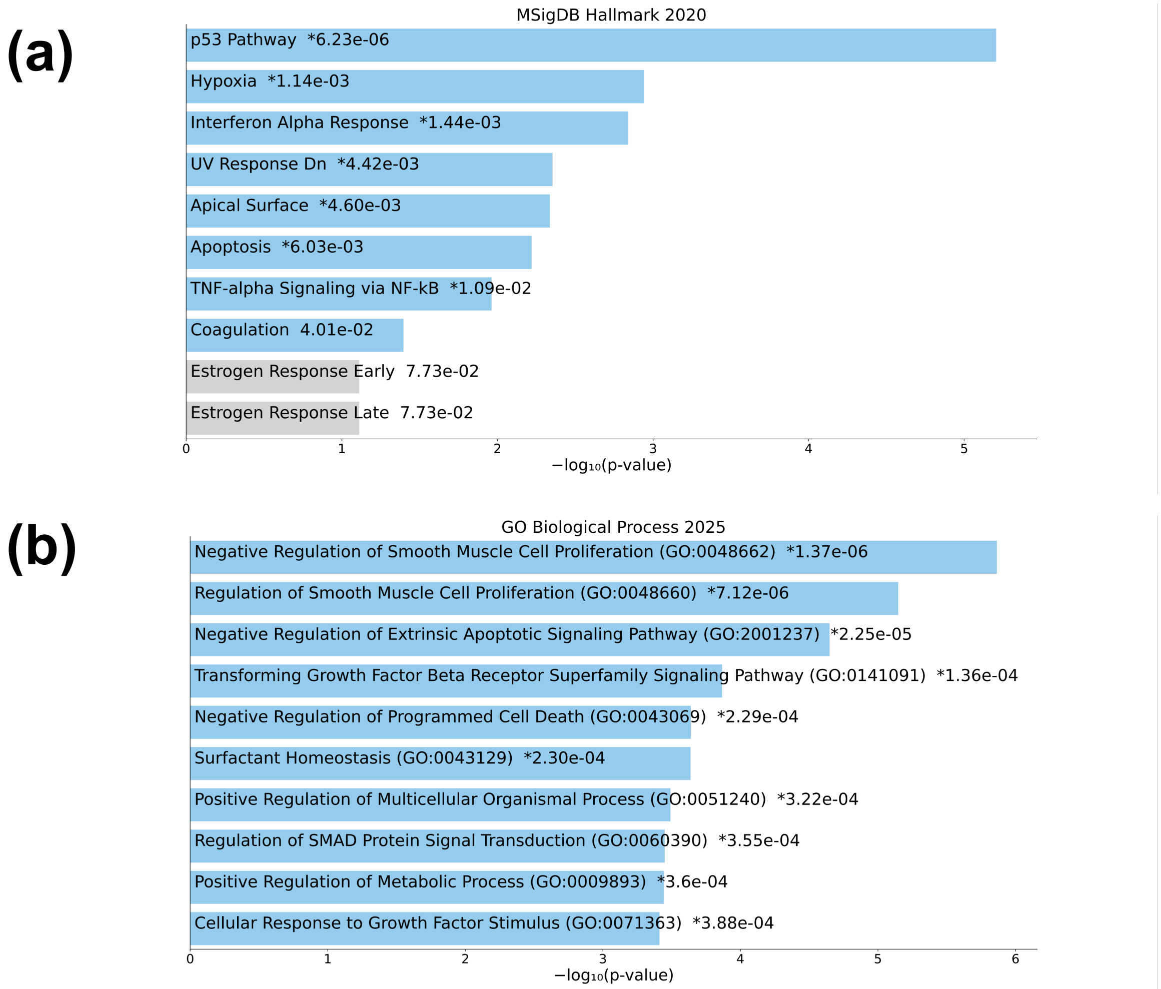

Figure S3. Enriched pathways associated with enzalutamide-docetaxel overlapping differentially expressed gene set. (**a**) Bar plots showing *p* values for the 10 most statistically significant pathways associated with the 93 DEGs corresponding to cross-resistance in the Human Molecular Signatures Database (MSigDB). (**b**) Bar plots showing the *p* values for the 10 most statistically significant pathways associated with the 93 DEGs corresponding to cross-resistance in the Gene Ontology (GO) Biological Process database.

Table S1. Gene pathways found to be significant in MSigDB Hallmark 2020

MSigDB Hallmark 2020			
**Pathway**	**p-value**	**q-value**	**Genes**
p53 Pathway	0.000006	0.000162	[VAMP8, DRAM1, ITGB4, PLK2, HMOX1, KLF4]
Hypoxia	0.00114	0.012508	[SELENBP1, BNIP3L, SRPX, HMOX1]
Interferon Alpha Response	0.001443	0.012508	[OAS1, IFI35, GBP2]
UV Response DNA	0.004425	0.02394	[ID1, PPARG, TGFBR2]
Apical Surface	0.004604	0.02394	[SRPX, HSPB1]
Apoptosis	0.006032	0.026138	[BNIP3L, HSPB1, HMOX1]
TNF-alpha Signaling via NF-kB	0.010906	0.040508	[DRAM1, PLK2, KLF4]
Coagulation	0.040149	0.130485	[ACOX2, ANG]

Table S2. Gene pathways found to be significant in GO Biological Process 2025.

GO Biological Process 2025			
**Pathway**	**p-value**	**q-value**	**Genes**
Negative Regulation of Smooth Muscle Cell Proliferation (GO:0048662)	0.000001	0.000875	[BMP4, HMOX1, ANG, PPARG]
Regulation of Smooth Muscle Cell Proliferation (GO:0048660)	0.000007	0.002278	[BMP4, HMOX1, ANG, PPARG]
Negative Regulation of Extrinsic Apoptotic Signaling Pathway (GO:2001237)	0.000022	0.004793	[BMP4, TMBIM1, HMOX1, KLF4]
Transforming Growth Factor Beta Receptor Superfamily Signaling Pathway (GO:0141091)	0.000136	0.021732	[BMP4, ID1, PPARG, TGFBR2]
Negative Regulation of Programmed Cell Death (GO:0043069)	0.000229	0.024551	[BNIP3L, PLK2, HSPB1, HMOX1, ANG, TXNDC5]
Surfactant Homeostasis (GO:0043129)	0.00023	0.024551	[TMEM63A, OAS1]
Positive Regulation of Multicellular Organismal Process (GO:0051240)	0.000322	0.024849	[BMP4, VAMP8, EPAS1, LCN2, PPARG, TGFBR2]
Regulation of SMAD Protein Signal Transduction (GO:0060390)	0.000355	0.024849	[BMP4, PPARG, TGFBR2]
Positive Regulation of Metabolic Process (GO:0009893)	0.00036	0.024849	[OAS1, EPAS1, LCN2, TGFBR2]
Cellular Response to Growth Factor Stimulus (GO:0071363)	0.000388	0.024849	[BMP4, HSPB1, KLF4, TGFBR2]

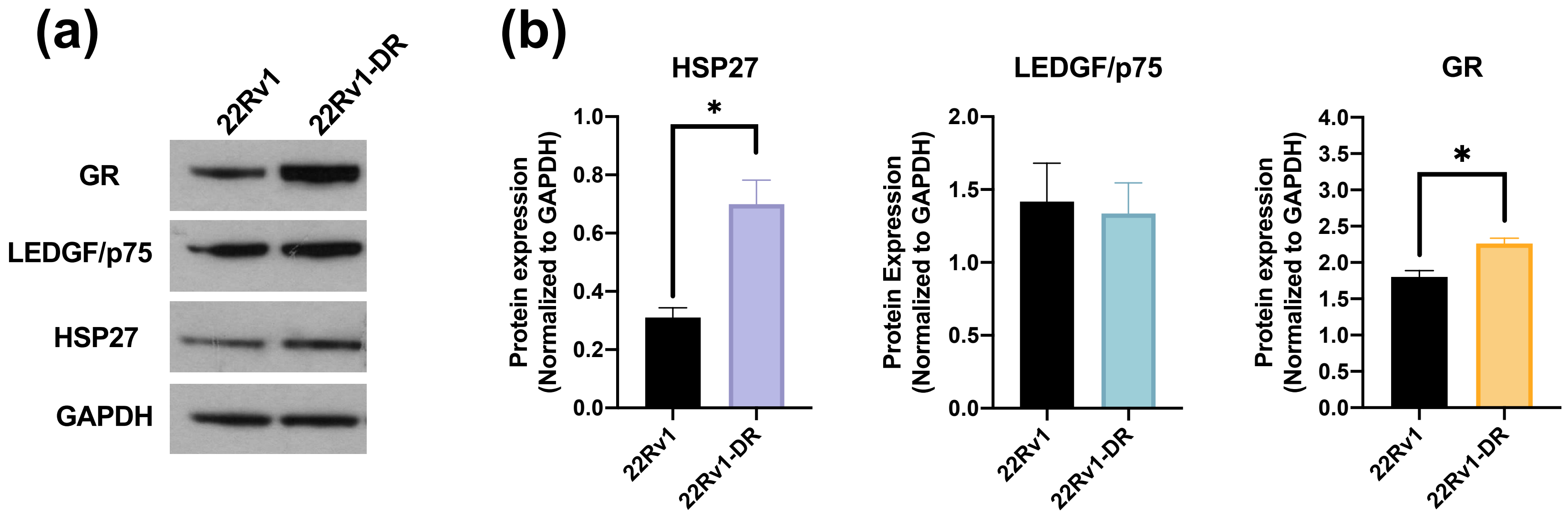

Figure S4. HSP27 is upregulated in docetaxel-resistant 22Rv1 cells. (a) Representative immunoblots and (b) quantification HSP27, LEDGF/p75, and GR protein expression. Quantified band values for HSP27, LEDGF/p75, and GR were obtained with ImageJ software and plotted as relative protein expression normalized to GAPDH. Statistical analyses were performed using unpaired *t* tests comparing sensitive cell lines to their resistant counterpart. Error bars represent mean +/− SEM from at least 4 independent experiments for each cell line. *p < 0.05, **p < 0.01, ***p < 0.001, ****p < 0.0001.

Supplementary Figure S5

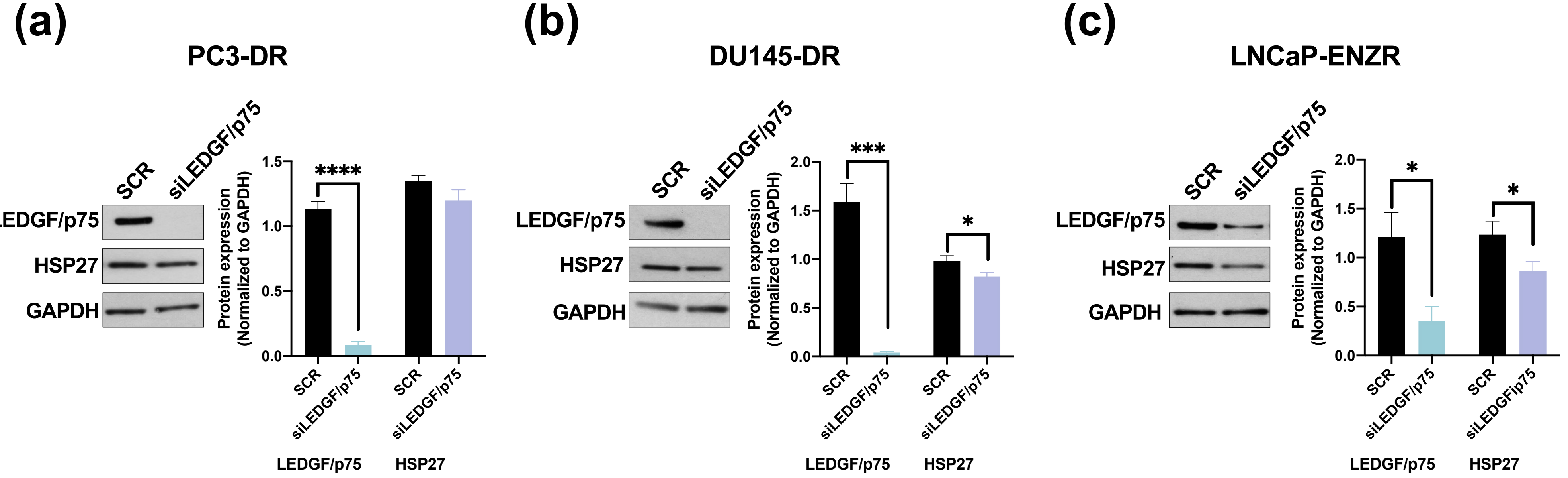

Figure S5. Silencing LEDGF/p75 leads to cell line-specific decrease in HSP27 protein expression levels in docetaxel- and enzalutamide-resistant PCa cells. DTX-resistant PCa cell lines PC3-DR (a), DU145-DR (b), and ENZ-resistant LNCaP-ENZR (c) were transfected with siRNA specific for LEDGF/p75 or scrambled negative control oligos (SCR) for 72 h, leading to significant HSP27 downregulation in DU145-DR and LNCaP-ENZR cells. Quantified band values for LEDGF/p75 and HSP27 were obtained with ImageJ software and plotted as relative protein expression normalized to GAPDH. Statistical analyses were performed using unpaired t tests comparing SCR to siLEDGF samples. *p < 0.05, **p <0.01. Error bars represent mean +/− SEM from at least 3 independent experiments for each cell line.

Supplementary Figure S6

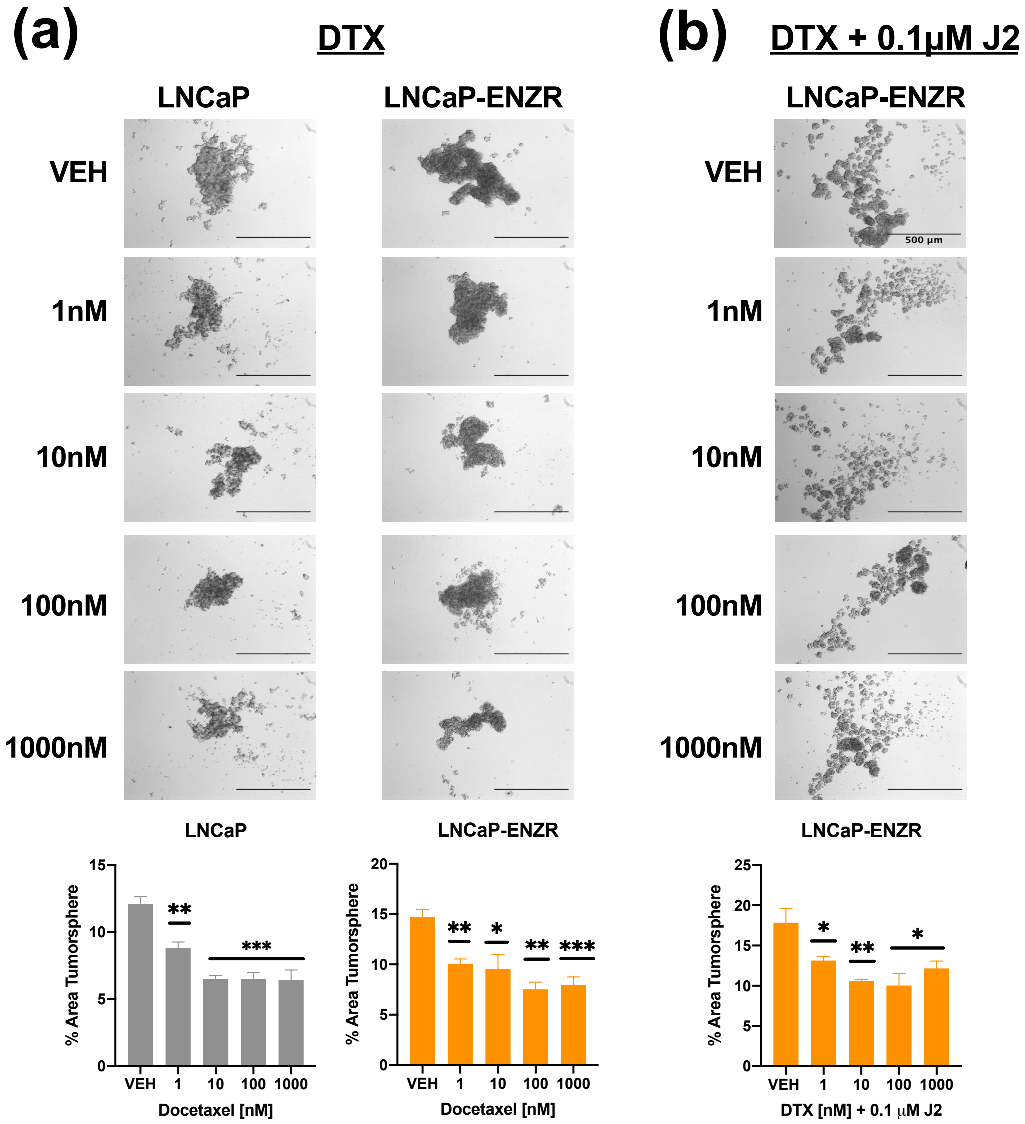

Figure S6. Inhibition of HSP27 in combination with docetaxel reduces tumorsphere formation in enzalutamide-resistant PCa cells. LNCaP and LNCaP-ENZR cells were cultured in MammoCult media supplemented with various concentrations of DTX (1 nM, 10 nM, 100 nM, 1000 nM) (**a**), and combination of J2 (0.1 μM) with ENZ (0.1 μM, 1 μM, 10 μM, 50 μM) (**b**) for five days. DMSO was used as the vehicle control. Images were captured using an Olympus IX70 microscope at 4× magnification, with a scale bar of 100 μm applied to all representative images. Tumorsphere area was quantified from four images per condition for each cell line. Data are presented as the mean ± SEM from three independent experiments. Statistical significance is indicated as follows: *$p < 0.05$, **$p < 0.01$, ***$p < 0.001$, ****$p < 0.0001$.

Table S3. Patient Demographics

Patient Demographic					
**Cohort**	**Primary tumors**	**Metastatic Tumors**	**NOS**	**All**	**Median Age**
High	1447	630	10	2087	68.0
Low	1364	793	17	2174	69.0
